# Online games training aging brains: limited transfer to cognitive control functions

**DOI:** 10.3389/fnhum.2012.00221

**Published:** 2012-08-17

**Authors:** Jesse van Muijden, Guido P. H. Band, Bernhard Hommel

**Affiliations:** Leiden Institute for Brain and Cognition, Leiden University Institute of PsychologyLeiden, Netherlands

**Keywords:** videogames, cognitive control, far transfer, cognitive enhancement, aging

## Abstract

The prevalence of age-related cognitive decline will increase due to graying of the global population. The goal of the present study was to test whether playing online cognitive training games can improve cognitive control (CC) in healthy older adults. Fifty-four older adults (age 60–77) played five different cognitive training games online for 30 min a day over a period of seven weeks (game group). Another group of 20 older adults (age 61–73) instead answered quiz questions about documentaries online (documentary group). Transfer was assessed by means of a cognitive test battery administered before and after the intervention. The test battery included measures of working memory updating, set shifting, response inhibition, attention, and inductive reasoning. Compared with the documentary group, the game group showed larger improvement of inhibition (Stop-Signal task) and inductive reasoning (Raven-SPM), whereas the documentary group showed more improvement in selective attention (UFoV-3). These effects qualify as transfer effects, because response inhibition, inductive reasoning and selective attention were not targeted by the interventions. However, because seven other indicators of CC did not show benefits of game training and some of those that did suffered from potential baseline differences, the study as a whole provides only modest support for the potential of videogame training to improve CC in healthy older adults.

## Introduction

The proportion of people over age 65 is steadily increasing worldwide (United-Nations, 2010). Given that cognitive functions decline with age (Meijer et al., 2009), age-related cognitive decline is becoming increasingly prevalent. Although there is a variety of effects of healthy old age on cognition, those on cognitive control (CC) functions have the most ubiquitous consequences (Burgess et al., 1998), as they are relevant for the selection and integration of information (Wild-Wall et al., 2011) and for dealing with novel situations that call for a deviation from automatized behavioral routines (Kramer et al., 1994; Wild-Wall et al., 2011). Impaired CC can therefore have serious consequences for the independence and quality of life of older adults. Fortunately, cognitive plasticity is preserved even at a very old age (Singer et al., 2003; Buschkuehl et al., 2008), so with the right interventions it seems feasible to reduce the dependence on caregivers and improve the quality of life in old adulthood.

Videogames have been recognized as a powerful tool for cognitive enhancement (Green and Bavelier, 2008). Indeed, positive effects of playing videogames on CC in old adults have been demonstrated (Basak et al., 2008; Peretz et al., 2011; Nouchi et al., 2012). Recently, however, a large-scale online study of videogame training among adults of all ages failed to show transfer of proficiency from trained tasks to untrained probe tasks (Owen et al., 2010). Although it successfully demonstrated that cognitive training is not a panacea, there is a risk that Owen et al.'s conclusions prematurely discredit videogame training, particularly in view of previous positive findings and the great potential it holds for buffering cognitive aging (Basak et al., 2008). Therefore, the current study addressed the need to clarify which CC functions can be enhanced by game training in healthy older adults, and whether there is transfer between the trained and untrained functions.

The study strictly followed methodological recommendations from the literature. First, an active control group was included in the experimental design to match the amount of computer use, adherence to a training schedule, and expectancy effects (Klingberg, 2010) to an extent that cannot be achieved by means of a waiting list control group. These participants watched documentaries and answered quiz questions online (Dustman et al., 1992). Second, we tested older adults on a series of CC tests before and after a substantial intervention (Klingberg, 2010), amounting to up to 49 videogame or documentary sessions. Transfer to cognitive domains subjected to training as well as transfer to untrained cognitive domains was assessed. The choice of transfer tasks was based on Miyake et al.'s (2000) taxonomy of CC functions as validated by latent variable analysis and, in contrast to Owen et al. (2010), the pretest and post-test measurements were taken under standardized laboratory conditions. Finally, the design of the online videogame training program was optimized according to recommendations of Green and Bavelier (2008). That is, the stimulus variability was high, difficulty levels of the games were continuously adapted to performance, and feedback and motivational messages were provided frequently. The design is also in accordance with empirically based recommendations for optimal learning as defined outside the context of game research. For example, in order to attain a higher level of performance large amounts of deliberate practice are required (Ericsson et al., 1993). The 49 times 30 min assigned in the current study will not raise the proficiency to expert levels, but it is a relatively long series in comparison with other game studies [e.g., 20 times 15 min in Nouchi et al. (2012); 24 times 10 min in Owen et al. (2010); 15 times 90 min in Basak et al. (2008)]. Typically deliberate practice is considered to be an effortful activity that can be sustained only for a limited time. However, not only are sessions limited to 30 min per day, the game context serves to reduce this burden and maintain motivation. Furthermore, as Schmidt and Bjork (1992) have reviewed, retention of learning benefits from the mixing of the training tasks, variability of the context, and relatively high task difficulty that were all present in the current study.

### Cognitive control

CC functions (Botvinick et al., 2001)—also referred to as executive functions (Miyake et al., 2000)—configure other cognitive functions for the performance of the task at hand. CC can be employed for biasing perceptual channels, actions, and memory representations on the basis of a task set. Because CC is involved in a wide variety of specific tasks and contexts, improving CC potentially buffers effects of cognitive aging. Miyake et al.'s (2000) taxonomy of CC functions was adopted in the current study, because it is widely accepted and empirically validated, and because it has proved to be valuable in analyzing age effects (Salthouse et al., 2003; Fisk and Sharp, 2004; Huizinga et al., 2006). The taxonomy describes CC as emerging from three distinct cognitive processes: switching between attentional sets or task sets (shifting), monitoring and updating information in working memory (updating), and inhibiting habitual, automatic, or prepotent responses (inhibition). Cognitive tests loading on these factors were included in the test battery used in the current study. In addition, selective and divided attention was assessed, to accommodate taxonomies of CC based on attentional processes (Posner and DiGirolamo, 1998).

CC takes a long time to fully develop in the course of childhood and eventually declines in the course of late adulthood (Zelazo et al., 2004; Kray et al., 2008). Decline of CC in old adults has been observed in both cross-sectional and longitudinal studies. Compared to younger adults, maintenance, and coordination of two alternating task sets in working memory (Mayr et al., 1996; Salthouse et al., 1998; Kray and Lindenberger, 2000; Kray et al., 2008), inhibitory control (Coubard et al., 2011) and divided and selective visual attention (Edwards et al., 2006) are impaired in healthy older adults. Longitudinal data from the Maastricht Aging Study (Meijer et al., 2009), the Berlin Aging Study (Lindenberger and Ghisletta, 2009), and the Advanced Cognitive Training for Independent and Vital Elderly study (Tucker-Drob, 2011) support the notion that the full range of CC functions declines with age. A recent analysis of longitudinal data from the Victoria Longitudinal Study (Macdonald et al., 2011) revealed that the rate of cognitive decline does increase with age, but remains slow and steady until the end of life. In the context of a graying global population, these developmental trends are alarming, because impaired CC is associated with impaired functioning in daily life (Burgess et al., 1998).

### Plasticity of cognitive control functions

According to the cognitive-enrichment hypothesis (Hertzog et al., 2009), the trajectory of cognitive development across the life span is not fixed. Although the trajectory of cognitive development at old age is largely determined by a lifetime of experiences and environmental influences, there is potential for discontinuity in the trajectory given a change in cognition-enriching behaviors. The cognitive-enrichment hypothesis is corroborated by ample evidence for plasticity—i.e., the potential for improvement of ability as a consequence of training (Denney, 1984)—of CC in the elderly population. Improvements of updating (Baron and Mattila, 1989; Buschkuehl et al., 2008; Dahlin et al., 2008), as well as shifting (Sammer et al., 2006; Bherer et al., 2008) and inhibition (Davidson et al., 2003; Karbach and Kray, 2009) in the population of older adults have been reported. In addition, selective attention (Ball et al., 2007) and inductive reasoning (Schmiedek et al., 2010) can be improved in older adults.

The virtue of a cognitive-training technique depends on the generalization—or transfer—of training to untrained tasks (Klingberg, 2010). Different degrees of transfer can be distinguished. Improvement within the same cognitive domain as subjected to training, assessed using different stimuli, and requiring a different response than the training task, is the minimal degree of transfer that can occur. This type of transfer is referred to as near transfer. Improvement of abilities in other cognitive domains than the cognitive domain subjected to training is referred to as far transfer.

Videogames are considered to provide an ideal context for cognitive enrichment (Achtman et al., 2008; Green and Bavelier, 2008). The characteristics of videogames presumed to facilitate transfer are their motivating nature, frequent presentation of feedback, precise reinforcement schedules, and stimulus variability (Gee, 2007). As a result of their entertainment value, videogames maintain the motivation to engage in practice for much longer than monotonous laboratory tasks or traditional training programs. Frequent feedback supports motivation and is also important for conditioning the desired level of performance. When the difficulty level of the game is continuously adapted to the performance, players will constantly be challenged at the limits of their ability. It is in particular the phase of skill-acquisition that calls for CC, whereas continued performance at a mastered level is associated with automatization and release of CC resources (e.g., Shiffrin and Schneider, 1977; Logan, 1988). Furthermore, small increments of difficulty level maximize the proportion of successful experiences with the game. Stimulus variability also plays an important role in training CC, because it helps to generalize learnt cognitive skills to multiple stimulus contexts.

Transfer of videogame interventions to CC has, however, not been demonstrated consistently. Owen et al. (2010), for instance, demonstrated that playing computerized cognitive training games like Nintendo's® Dr. Kawashima's Brain Training™ was not more beneficial for CC functions than answering general knowledge questions online. Because the sample of participants in Owen et al.'s study was very heterogeneous and included both young and old adults, it is well possible that improvements of cognitive test performance were attenuated in young adults due to ceiling performance at pretest. This could have obscured possible transfer of training in the subsample of older adults. The notion that sample heterogeneity can confound the observed effect of videogame training substantially is corroborated by Feng et al. (2007). They found no effect of playing action videogames on spatial attention in a sample of young adults. However, separate analyses of the effect in males and females revealed that females did actually benefit from playing videogames. In addition, Owen et al.'s participant sample was very heterogeneous with respect to training adherence, so participants who completed only two training sessions could have had a negative impact on aggregated training outcomes. Another aspect of Owen et al.'s study that makes the observed absence of transfer difficult to interpret is that transfer was assessed using a test battery comprising only four cognitive tests, three of which were measures of working memory capacity.

Ackerman et al. (2010) demonstrated that sample heterogeneity cannot account for Owen et al.'s (2010) findings. They found that playing cognitive training games (Nintendo® Wii™ Big Brain Academy™) does not benefit cognitive abilities to a greater extent than reading assignments do, in a homogeneous sample of healthy older adults on a relatively fixed and extensive training schedule. Moreover, a broader assessment of cognitive abilities of interest was made than in Owen et al.'s study. Still, Ackerman et al. focused predominantly on reasoning ability and perceptual processing speed, while a large share of the videogames under study taxed working memory updating and the large variety of videogames probably stimulated participants' attention and task set shifting. Inclusion of transfer tasks gauging working memory updating and set shifting in Ackerman et al.'s study could have led to different conclusions regarding transfer of playing cognitive training games.

Conversely, there is also some evidence against Owen et al.'s (2010) and Ackerman et al.'s (2010) pessimistic conclusions regarding the beneficial effects of playing videogames on CC functions. Namely, Peretz et al. (2011) found a larger improvement of visuospatial working memory, visuospatial learning, and focused attention after playing Cognifit Personal Coach® cognitive training games than after playing conventional videogames that were matched for intensity, in a sample of older adults. Even though there is some theoretical overlap in the cognitive functions assessed by Peretz et al. and Owen et al. and Ackerman et al., the specific cognitive tests used to assess transfer in these studies was different. It is conceivable that some cognitive tests are more sensitive to transfer effects than others, which might explain the discrepant results of these studies.

Furthermore, playing videogames not specifically designed for cognitive training can also improve CC functions in older adults. Basak et al. (2008) demonstrated that playing a particular complex 3-D real-time strategy game (Rise of Nations) was associated with greater improvements of shifting, updating, and inductive reasoning than observed in the control condition. It must be noted that the control group in this study was a no-contact control group, so it is not certain to what extent the observed improvements in the videogame group are attributable to placebo-effects. Nevertheless, the improvements of CC in this study were larger than practice effects due to repeated exposure to the same cognitive test.

It has been argued that failures to demonstrate far transfer of playing cognitive training games in the population of older adults may be due to a general age-related decrease of the extent to which learning transfers to untrained abilities (Ackerman et al., 2010). This assertion is supported by Ball et al.'s (2002) finding that cognitive strategy training programs for improving memory, processing speed and reasoning, respectively, were associated with improvements within the trained cognitive domain but not with far transfer to untrained cognitive abilities of older adults. In contrast, however, far transfer of practicing basic cognitive tests has been reported repeatedly in the cognitive aging literature (Mahncke et al., 2006; Uchida and Kawashima, 2008; Karbach and Kray, 2009; Smith et al., 2009). Brain training games like Nintendo's® Dr. Kawashima's Brain Training™ share many task components of basic cognitive laboratory tasks and videogames have several additional characteristics facilitating transfer (Green and Bavelier, 2008). Therefore, it is reasonable to expect that transfer of computerized cognitive training games in the population of older adults is replicable.

### Current study

It is difficult to reconcile inconsistent findings pertaining to the effect of playing cognitive training games on cognition (Ackerman et al., 2010; Owen et al., 2010; Peretz et al., 2011), because the methodological differences between these studies are substantial. More research is required to elucidate what aspects of brain training games facilitate transfer to untrained cognitive abilities. Hence, the aim of the present study was to test whether playing brain training games does transfer to different measures of CC in healthy older adults. An online brain training game intervention (Owen et al., 2010; Peretz et al., 2011) was compared to an intervention requiring participants to watch documentaries and answer quiz questions online (Dustman et al., 1992). Transfer was assessed by comparing performance on a battery of cognitive tests before and after the intervention. Taking into account that some cognitive tests may be more sensitive to transfer effects than others, several measures of updating, shifting, and inhibition were included in the test battery. To avoid transfer effects beyond the currently used taxonomy of CC from being overlooked, measures of selective attention and inductive reasoning were also included in the test battery. While improvement on CC measures is to be expected in both groups, the crucial test is whether the improvement in the videogame condition exceeds that of the documentary condition.

Two of the games—Firemen and Falling Bricks—were specifically designed to tax updating. In these task, the speed by which participants had to update their working memory content was pushed to the limits. Two of the games were designed to tax shifting; Giving Change and Firemen. In both games, performance required switching between addition and subtraction. As for Anagrams and Telling Time, these were chosen because it is plausible that these tasks put a high demand on CC and working memory maintenance, and because they are part of the Nintendo set. Based on the task demands of these games and evidence for near transfer of cognitive training games (Ball et al., 2002; Peretz et al., 2011), near transfer of training to updating and shifting was expected. Measures of inhibition, reasoning, and selective attention were included in the cognitive test battery, but these cognitive functions were not primarily targeted by the training program. Thus, possible improvements thereof could be considered a demonstration of far transfer. Far transfer can be expected based on ample evidence for far transfer of cognitive training in the population of older adults (Mahncke et al., 2006; Uchida and Kawashima, 2008; Karbach and Kray, 2009; Smith et al., 2009). However, there is also evidence to suggest that cognitive training games are perhaps too different from transfer task for transfer of learning to occur in older adults (Ackerman et al., 2010).

## Materials and methods

### Participants

Ninety-two participants were recruited through advertisements in a local newspaper and on the internet. Ten participants prematurely withdrew from the study, six in the documentary group (24%) and four in the videogame group (7%, χ^2^_(1)_ = 5.2, *p* < 0.05). Another eight participants could not complete the intervention due to technical issues, time constraints, or medical problems. Two additional participants with a Mini Mental State Examination (MMSE; Folstein et al., 1975) score lower than 27 out of 30 points were excluded from the analyses. Data of the remaining 72 participants were analyzed. All these participants were community dwelling citizens, free of neurological deficits or traumatic brain injury, and cognitively healthy according to prevalent MMSE norms. Prior to the study, participants in the videogame group did not differ from participants in the documentary group with respect to age, years of education, Raven IQ (Raven, 1938), MMSE score and previous computer game experience (Table 1). Full participation was rewarded with €100. All participants gave their informed consent prior to participation. The study was approved by the Ethical Committee of the Institute of Psychology, Leiden University.

**Table 1:** **Distribution of males and females across conditions and mean (SD) age, years of education, MMSE score, and Raven SPM IQ in each condition**.

	**Experimental group**	**Documentary group**		
Gender	*n*_male_ = 25	*n*_male_ = 15	χ^2^_(1)_ = 5.7	*p* < 0.05
	*n*_female_ = 28	*n*_female_ = 4		
Age	67.8 (3.8)	67.2 (3.4)	*t*_(70)_ = 0.7	*p* > 0.05
Years of education	13.2 (4.4)	11.8 (3.4)	*t*_(70)_ = 1.2	*p* > 0.05
MMSE	28.8 (1.2)	28.9 (0.9)	*t*_(70)_ = −0.2	*p* > 0.05
Raven SPM IQ	115.7 (12.3)	120.1 (9.8)	*t*_(70)_ = −1.4	*p* > 0.05
Played videogames	25%	24%	χ^2^_(1)_ < 1	*p* > 0.05

### Materials

The online intervention programs were developed using Adobe Authorware 7 (©Adobe Systems Incorporated, 2011). Cognitive tests were programmed in E-prime 2.0 (©Psychology Software Tools, Inc., 2010). A PC with a 15″ CRT monitor with a refresh rate of 85 Hz was used for the administration of cognitive tests. Auditory stimuli were presented by means of headphones.

### Procedure

The experimental design was a randomized controlled trial. Consistent with the recommendations of (Boot et al., 2011), participants were told that the study compared two brain training interventions, without reference to either condition as the control or test condition. Participants in both groups were motivated to do well on the intervention, by means of the same set of motivational messages incorporated in the intervention programs. Participants in the videogame group played five randomly alternating videogames. The videogames were custom built, inspired by commercially available cognitive training games. Feedback on performance was presented after every response. The difficulty level of each game was raised or lowered depending on the performance in the preceding round of the respective game. Participants in the documentary group watched documentaries with a duration of approximately 30 min. A different documentary was presented every session. After watching a documentary, participants had to answer three to five multiple-choice quiz questions about the documentary. The same feedback stimuli as used in the videogames were presented after every response. Participants were instructed to complete one 30-min intervention session per day, every day of the week, for seven weeks, resulting in a total of up to 24.5 h of training. Participants who were unable to complete a session on one day were instructed to complete an extra session on another day. The intervention was available online, hosted on a faculty server. This enabled participants to complete the intervention program at home and it allowed the experimenters to track intervention compliance and performance.

All participants completed a cognitive test battery comprising nine cognitive tests before and after the intervention. In addition, participants were subjected to the MMSE and completed a general health questionnaire at pretest. The pre- and post-test assessments were conducted in the cognitive-psychology laboratory of Leiden University. Three different test sequences were devised and these were counterbalanced across participants. Each participant completed the test battery in the same order at pre- and post-test. The test battery took approximately two hours to complete. Participants were allowed to take a 10-min break after the first hour of testing.

### Videogames

The videogames were presumed to tax CC, as they required players to select and integrate information, manipulate working memory representations, and switch between task sets.

#### Anagrams

**Figure 1:**
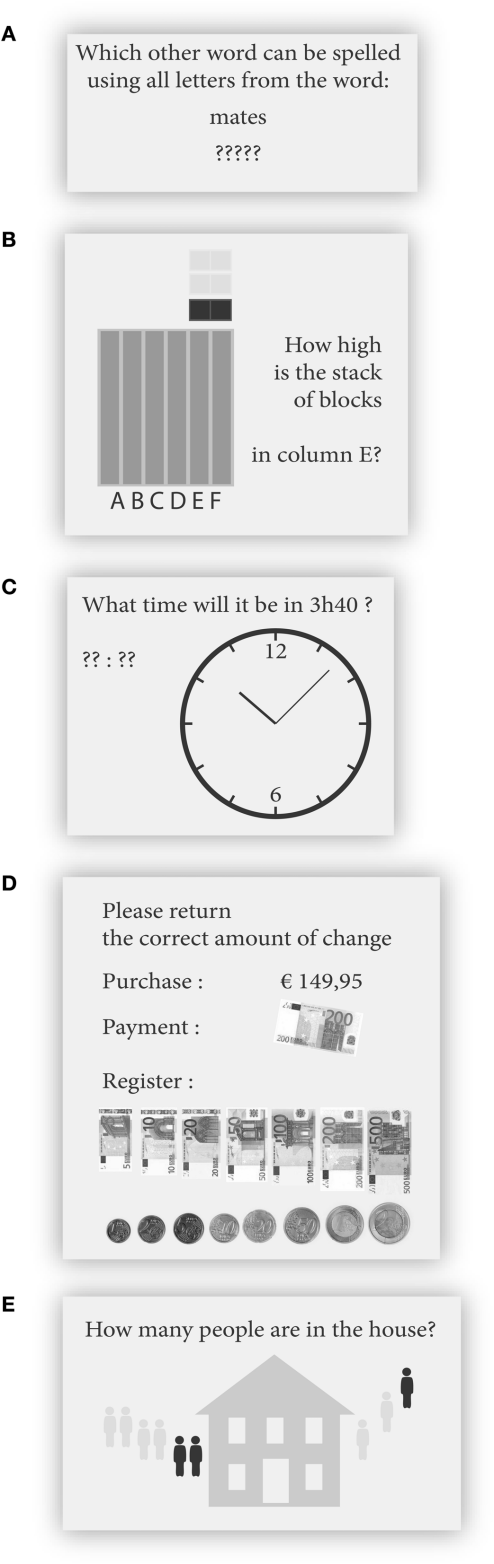
**Impression of the games constituting the game intervention**. **(A)** Anagrams, **(B)** Falling Bricks, **(C)** Telling Time, **(D)** Giving Change, **(E)** Firemen. All text was presented in Dutch.

In the Anagrams game (Figure 1A) a different string of letters was presented every game round. Players were instructed to spell a new word using all of the presented letters. At the lowest difficulty level anagrams were three letters long. The most difficult anagrams were nine letters long. As players advanced, the length of the presented letter strings increased.

#### Falling bricks

In the Falling Bricks game (Figure 1B) an animation of bricks falling down behind an occluding rectangle was presented. The occluding rectangle was subdivided into several columns. After the animation, players had to indicate how high the stack of bricks in one cued column was. As players advanced, the total number of falling bricks and the number of columns constituting the occluding rectangle, increased. The number of columns to monitor ranged from 1 to 10. The number of falling bricks ranged from 1 to 11.

#### Telling time

In the Telling Time game (Figure 1C) an analog clock was presented. Players were instructed to indicate what time it would be after a variable number of hours and minutes, given the current time depicted on the clock. As players advanced, the complexity of the time addition increased. At the lowest difficulty level the time difference was 3 h at most. At the highest level, the time difference was 24 h at most.

#### Giving change

In the Giving Change game (Figure 1D) players were presented with a price to be paid and a payment that has been made. The player's task was to return change by clicking the optimal combination of bills and coins. As players advanced, the presented prices and payments increased. At the lowest difficulty level the presentation time of the price and the change the player had already returned, were presented for an infinite amount of time. In addition, the highest price was €5 and players were allowed to return five coins and bills more than minimally necessary to make the correct change. At the highest difficulty level the prices were presented for only 3 s, no online feedback was provided regarding the amount already returned, the maximum payment was €500 and no more coins or bills than necessary were allowed to be returned.

#### Firemen

In the Firemen game (Figure 1E) an animation of several groups of stick figures moving into or out of a house was presented. Groups of one to five stick figures were presented at a time. Players were required to keep track of the number of stick figures inside the house. After the animation, players were prompted to type in the remaining number of stick figures residing in the house. As players advanced, stick figures walked into and out of the house with greater frequency and in larger numbers.

### Cognitive test battery

#### Mini mental state examination

The MMSE is the most widely used assessment of global cognitive function (Folstein et al., 1975). It is often used to screen for dementia or monitor its progression. A Dutch version of the test was used.

#### Stroop color-word test

In a computerized version of the Stroop Color-Word Test (Stroop, 1935) participants were instructed to ignore a visually presented Dutch color name (“rood,” “blauw,” or “groen”) corresponding to either red, blue, or green and identify the font color of the stimulus (red, blue, or green) as quickly as possible by choosing a keyboard key (“C,” “V,” or “B”), each corresponding to a stimulus color. The mapping of stimulus colors to buttons was balanced across participants. All possible combinations of stimulus color and color name were presented 20 times in random order. The test consisted of four blocks of 45 trials, separated by short breaks. A trial started with the 1500 ms presentation of a central fixation cross. Subsequently, a stimulus was presented centrally for a maximum duration of 3000 ms or until a response was detected. The response-stimulus interval (RSI) was randomized (600–800 ms). Only reaction times (RT) associated with correct responses were analyzed. The mean RT difference between the incongruent and congruent conditions was used as a dependent variable, which is assumed to measure inhibition (Miyake et al., 2000).

#### Stop-signal test

In the Stop-Signal Test (Logan et al., 1984), each trial started with a fixation cross presented for 250 ms, followed by an “O” or “X” in the center of the screen lasting for 2000 ms, or until a response was detected. Participants were instructed to indicate which of the two stimuli was presented by pressing one of two keyboard keys (“C” or “N”). Stimulus-response mappings were balanced across participants. In addition, participants were instructed to try to withhold their response if they heard a computer-emitted tone on 33% randomly selected trials, but not to slow down in anticipation of stop signals. Participants practiced nine trials before the actual experiment started. The experiment consisted of 3 blocks of 36 trials. The stimulus onset asynchrony (SOA) of the visual stimulus and the auditory stop signal started at 30 ms and varied depending on stop success following a staircase algorithm aiming at 50% accuracy, with step sizes of 30 ms and a maximum SOA of 700 ms. Stop-signal reaction time (SSRT), defined as the difference between median RT in GO-trials and mean SOA (Band et al., 2003) was used as dependent variable. To obtain a reliable measure of SSRT, the analysis was limited to participants with 10–90 percent correct inhibition and at least 60 percent accuracy on nonsignal trials. The Stop-Signal Test is considered a measure of inhibition (Logan et al., 1984).

#### Counting span

The Counting Span task (Conway et al., 2003) required participants to count the number of blue circles within serially presented stimulus arrays. After a series of stimulus arrays was presented, participants were prompted to recall the total number of blue circles in each stimulus array, in the correct order. The stimulus arrays also contained distracters with either the same shape or the same color as the target stimulus. Participants first practiced four trials, each consisting of a series of two stimulus arrays. Trials in the subsequent experimental block could consist of two to five stimulus arrays. All trial types were replicated three times. The order of trial types was pseudo-randomized and stimulus presentation was self-paced. Participants were instructed to count the targets out loud, repeat the total number of targets out loud and press the spacebar on the keyboard to advance to the next stimulus array. After the last stimulus array, participants were prompted to type in the recalled number of targets in each stimulus array presented in the current trial. The total number of correctly recalled counts in the condition with highest memory load was used as dependent variable. The counting span task can be considered as a measure of updating (Schmiedek et al., 2009).

#### Mental counters

The Mental Counters task (Larson and Saccuzzo, 1989) required participants to keep track of multiple variable numbers. Each number to be updated was represented by a horizontal bar on the screen. The number started at a value of five and had to be increased or decreased whenever an “X” was presented above or below the bar, respectively, and should not be changed if an “?” was presented. The inter-stimulus interval was 1700 ms. After five or six updates, participants were prompted to enter the final value of each number at their own pace. The test consisted of 2 blocks of 10 trials. Participants had to keep track of two numerical representations in the first block and three numerical representations in the second block. The number of updates required was randomized across trials. The mean number of correct responses in the condition with three numbers was used as dependent variable. The mental counters task provides a measure of updating (Huizinga et al., 2006).

#### Useful field of view test

A divided attention and a selective attention subtest of the Useful Field of View Test (Edwards et al., 2005) were administered. In both subtests, participants were instructed to identify the shape of a briefly presented central car or truck stimulus and the location of simultaneously presented peripheral car stimulus. The peripheral target could appear at one of eight radial locations. In the selective attention subtest, the empty parts of the stimulus display were filled up with distracters (triangles). A fixation box was presented at the beginning of a trial. Next, both stimuli were presented simultaneously. The screen was filled with a white-noise visual mask immediately after stimulus presentation. Then, two response screens appeared consecutively, prompting for the identity of the central stimulus and the location of the peripheral stimulus by mouse clicks. The duration of stimulus presentation was determined by a staircase algorithm aiming at 75% accuracy. The duration of stimulus presentation associated with 75% accurate performance on the divided (UFoV2) and selective attention (UFoV3) subtest was used as dependent variable.

#### Raven standard progressive matrices

The Raven's Standard Progressive Matrices (Raven-SPM; Raven, 1938) consists of textural patterns and 3 × 3 matrices of figures from which one part is missing. Participant were required to indicate which of six or eight alternatives correctly completed the presented pattern. We used a shortened, computerized version of the Raven SPM (Keizer et al., 2010), consisting of either the 30 even or 30 odd items, with a time limit of 10 min. One subset was administered at pretest and the other at post-test in balanced order. Raven IQ scores corrected for age (Peck, 1970) were used as dependent variable. The Raven-SPM test provides a measure of inductive reasoning ability (Schmiedek et al., 2009).

#### Global-local switching test

In the Global-Local Switching Test (Huizinga et al., 2006), participants were required to respond to either the local or the global shape of a large square or rectangle consisting of small squares or rectangles. The size of these response alternatives displayed at the bottom of the screen indicated whether the participant was required to match the response to the local or global shape of the stimulus. The relevant size (global vs. local) was constant in two pure blocks, and varied randomly in two mixed blocks of 30 trials. The order of blocks and relevant stimulus dimensions was counterbalanced across participants. A practice block of eight trials preceded the actual experiment. At the beginning of each trial a central fixation cross was presented for 200–400 ms (randomized). The response alternatives were presented next. The stimulus was added to the display with a 500 ms delay. A trial ended after 4000 ms or when a response was detected. RSI was 500 ms. Switch cost was used as dependent variable (Karbach and Kray, 2009). Switch cost was defined as average RT difference between trials with switched versus repeated size instructions, within the mixed block. The Global-Local Switching Test provides a measure of shifting (Huizinga et al., 2006).

#### Smiling faces switching test

The Smiling Faces Switching Test (Huizinga et al., 2006) required participants to respond to either the emotional expression or gender of faces. The stimuli were simple line drawings of a male or female face with a happy or sad facial expression. Stimuli could appear in one of the quadrants of a 2 × 2 grid. The relevant stimulus dimension was determined by the row in which a stimulus was presented. The mapping of relevant stimulus dimensions on rows was balanced across participants. Trials were blocked in exactly the same fashion as in the Global Local Switching Test. Participants were instructed to respond by pressing the “Z” or “M” key. Each key was associated with one facial expression and one gender. Stimulus-response mappings were balanced across participants. At the beginning of each trial a central fixation cross was presented for 200–400 ms (randomized). Subsequently, the stimulus was presented for 4000 ms or until a response was detected. The RSI was randomized (200–400 ms). Switch cost was used as dependent variable (Span et al., 2004).

#### Test of attentional performance

The Test of Attentional Performance (Majer et al., 2004) requires participants to perform a visual discrimination task and an auditory 1-back task in parallel. In the visual task a 4 by 4 grid consisting of dots and crosses was presented on each trial. Subjects were instructed to press the “C” key on the keyboard if a square of crosses was formed on any four adjacent points on the grid. In the auditory 1-back task either a high-pitch (990 Hz) or low-pitch (660 Hz) tone was presented every trial. Subjects were instructed to press the “V” key on the keyboard if the currently presented tone had the same pitch as the tone presented on the previous trial. Participants were instructed to pay attention to both tasks at the same time and react as fast as they could while maintaining a high level of accuracy. Participants first completed three practice blocks consisting of six trials. In the first two practice blocks, the individual tasks were practiced in isolation. In the third practice block participants practiced the dual task. After the practice blocks, participants completed three experimental blocks consisting of 60 trials. The inter-stimulus interval was 2900 ms when no response was detected. The RSI was 800 ms. Although participants were instructed to perform both tasks in parallel, a target was never presented in both modalities simultaneously. The accuracy of target detection was used as dependent variable. The Test of Attentional Performance is considered to be a measure of divided attention (Majer et al., 2004).

## Results

### Videogame performance

The time spent on the intervention did not differ statistically between the videogame (*M* = 21.1 h, *SD* = 3.3) and the documentary group (*M* = 22.9 h, *SD* = 3.9; *t*_(71)_ = 2.0, *p* > 0.05). Participants reached increasingly higher levels in all the games. The use of statistical tests in analyzing game level progress would be misleading, however. Games are not suited to yield accurate capability scores for each session, for example because starting levels were deliberately easy to perform and multiple parameters changed with each successive level. Suffice it to note, therefore, that on average the participants eventually managed to solve anagrams of 7 letters (*SD* = 0.4), and monitored 6.5 (*SD* = 1.9) columns during 6.9 (*SD* = 2.0) updates in the Falling Bricks game. In the Fireman game, 9.4 (*SD* = 0.9) updates were made, with speeds of 753 ms (*SD* = 188 ms) per update. All participants were eventually able to complete the Telling Time and the Giving Change game at the highest difficulty level.

The subjective experience of the videogame and documentary intervention was not systematically assessed, but a surprisingly large proportion of participants left remarks about their experience of the intervention in the general exit questionnaire. Although these data are confounded by response bias, they still give some, albeit tentative, insight into the success of the intervention. Sixty-six percent of participants in the videogame group reported about how much they enjoyed the intervention, compared to 90% in the documentary group. Fifty-one percent of all participants and 77% of responding participants in the videogame group stated to have enjoyed the intervention. Thus, 23% of all participants in this condition indicated that they had not enjoyed the videogames without being inquired about it. In the documentary group, 79% of all participants and 88% of responding participants indicated that they enjoyed the intervention. Only 12% of all participants in this condition indicated that they had not enjoyed the documentaries without being inquired about it.

### Transfer

For the Stroop test, UFoV-3, Smiling Faces Switching Test and Global Local Switching Test, trials with RTs outside a 2 SD range were excluded for each participant. For each dependent variable, participants with individual mean scores outside a 3.5 SD range per group were excluded. Repeated-measures ANOVA with Intervention (videogame vs. documentary) as between-subject factor and Time (pre-test vs. post-test) as within-subject factor were conducted to analyze transfer of training to each dependent variable separately. The interaction effect of Intervention and Time was significant on SSRT, UFoV-3, and Raven IQ. These effects were small to medium sized (η^2^_*p*_ < 0.10). The results of all univariate analyses are summarized in Table 2, and mean scores are summarized in Table 3.

**Table 2:** **Results obtained from univariate ANOVA of the interaction effect of Intervention and Time on every dependent variable**.

**Test**	**Statistic**	**Significance**	**Effect size (η^2^_*p*_)**
Raven SPM	*F*_(1, 69)_ = 5.0	*p* < 0.05^*^	0.068
Stroop	*F*_(1, 67)_ < 1	*p* > 0.1	0.001
Stop-signal	*F*_(1, 54)_ = 5.2	*p* < 0.05^*^	0.087
Mental counters	*F*_(1, 69)_ < 1	*p* > 0.1	0.011
Counting span	*F*_(1, 68)_ < 1	*p* > 0.1	<0.001
Smiling faces	*F*_(1, 69)_ = 1.7	*p* > 0.1	0.024
Global local	*F*_(1, 66)_ < 1	*p* > 0.1	<0.001
TAP	*F*_(1, 68)_ < 1	*p* > 0.1	<0.001
UFoV-2	*F*_(1, 69)_ < 1	*p* > 0.1	<0.001
UFoV-3	*F*_(1, 68)_ = 4.3	*p* < 0.05^*^	0.059

* , p < 0.05.

**Table 3:** **Mean (SE) scores for the cognitive indices used, divided by group and test time**.

**Index**	**Videogame Group Mean (SE)**		**Documentary Group Mean (SE)**	
	**Pre-test**	**Post-test**	**Pre-test**	**Post-test**
IQ Raven SPM	116.4 (1.5)	119.4 (1.5)	120.1 (2.5)	116.8 (2.5)
Stroop	156.6 (18.7)	155.7 (15.5)	113.8 (31.5)	105.8 (26.0)
Stop-signal	326.2 (18.8)	249.8 (12.3)	245.6 (28.5)	239.9 (18.6)
Mental counters	1.67 (0.10)	2.05 (0.10)	1.82 (0.16)	2.05 (0.16)
Counting span	12.6 (0.3)	13.3 (0.3)	12.7 (0.5)	13.1 (0.5)
Smiling faces	365.6 (32.6)	343.7 (34.5)	286.1 (55.9)	368.2 (59.3)
Global local	83.5 (25.2)	61.8 (29.8)	87.9 (42.0)	118.9 (49.6)
TAP	0.91 (0.01)	0.93 (0.01)	0.93 (0.02)	0.94 (0.02)
UFoV-2	165.0 (18.6)	116.9 (14.8)	131.5 (30.8)	86.2 (24.4)
UFoV-3	276.1 (15.2)	261.5 (14.5)	273.3 (24.9)	208.3 (23.8)

See the main text for explanation of the indices.

**Figure 2:**
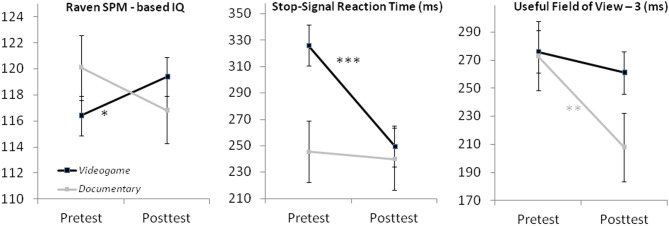
**Aggregate stop-signal RT, Raven IQ, and UFoV3 data at pre- and post-test in the game (black lines) and documentary (gray lines) condition**. Significant changes in performance from pre- to post-test within each condition are indicated by asterisks. ^*^p = 0.05, ^**^p < 0.05, ^***^p < 0.01.

As expected, the improvement of Stop-Signal task and Raven-SPM performance was larger in the videogame group than in the documentary group (Figure 2). The mean SSRT decreased from 326 ms at pretest to 250 ms at post-test in the videogame group. In the documentary group the mean SSRT decreased from 246 to 240 ms. The mean Raven IQ scores were above Peck's (1970) average norm scores in both groups at both assessments. In the videogame group, mean Raven IQ increased from 116 at pre-test to 119 at post-test, while mean Raven IQ decreased from 120 to 117 in the documentary group. However, contrary to expectations, the improvement of UFoV-3 performance was larger in the documentary group than in the videogame group. The videogame group improved from 276 ms at pretest to 261 ms at post-test, while the documentary group improved from 273 ms to 208 ms. The change of performance from pre-test to post-test on all dependent variables in the videogame group and the documentary group is illustrated in Figure 2.

The significant interaction effects were further analyzed by means of simple effect analyses of the difference between pre-test and post-test performance within each intervention group (Figure 2 and Table 4). The improvement of SSRT was significant in the videogame group, while there was no significant change of SSRT over time in the documentary group. The improvement of SSRT in the videogame group can be considered a large effect (Cohen, 1992). The increase of Raven IQ in the videogame group was marginally significant, as was the decrease in Raven IQ in the documentary group. The improvement of UFoV-3 was only significant in the documentary group and was also large.

**Table 4:** **Significance of simple effects of time in each intervention condition**.

**Test**	**Condition**	**Statistic**	**Significance**	**Effect size (η^2^_p_)**
Raven SPM	Game	*F*_(1, 51)_ = 3.9	*p* = 0.05	0.071
	Documentary	*F*_(1, 18)_ = 2.7	*p* > 0.1	0.128
Stop-signal	Game	*F*_(1, 38)_ = 17.6	*p* < 0.001	0.316
	Documentary	*F*_(1, 16)_ < 1	*p* > 0.1	0.004
UFoV-3	Game	*F*_(1, 50)_ = 1.3	*p* > 0.1	0.025
	Documentary	*F*_(1, 18)_ = 10.4	*p* < 0.01	0.367

### *Post-hoc* analyses

There were two issues that required further elaboration to fully appreciate the value of the transfer effects. First, the analysis of the participants' background characteristics revealed that the videogame and documentary group were significantly unbalanced in terms of gender composition. Considering Feng et al.'s (2007) finding that gains in spatial attention due to playing action videogames were larger for women than for men, the transfer effects observed in the present study may be confounded by the dissimilar gender composition of the videogame group and the documentary group. Three-way repeated-measures ANOVA including Gender (male vs. female) and Intervention (videogame vs. documentary) as between-subject factors, and Time (pre-test vs. post-test) as within-subject factor were conducted to falsify this alternative explanation of the observed transfer effects. The Gender X Intervention X Time interaction effects on SSRT, UFoV-3, and Raven IQ were all not significant (all *F*s < 2, *p*s > 0.2), indicating that there was no difference between men and women regarding the differential patterns of improvement of these cognitive functions in the videogame group and the documentary group.

Second, based on the patterns in the aggregate data, it could be argued that some of the significant interaction effects can be explained by anomalous performance at baseline in either one of the groups (Boot et al., 2011). To address this issue, *post-hoc t*-tests of the difference between the videogame and documentary condition regarding SSRT, Raven IQ, and UFoV-3 at pretest were performed. Only SSRT performance was significantly different between groups at pretest (*t*_(54)_ = 2.4, *p* < 0.05; other *p*s > 0.2). Because this SSRT analysis depended on a strict selection of participant performance, we looked for background differences between participant who had and those who had not been included in the analysis. There were no differences in age, MMSE, education, participation, gender composition, or Raven scores during pretest (all *p* > 0.1), so there is no reason to doubt whether the restricted sample is representative for the larger group.

## Discussion

The goal of the present study was to test whether playing online cognitive training games effectively benefits CC in a healthy elderly sample. An online cognitive training game intervention was compared to an intervention requiring participants to watch documentaries and answer quiz questions online. Based on the results of a similar study (Peretz et al., 2011) and ample evidence for far transfer of practicing basic cognitive tests to CC of older adults (Mahncke et al., 2006; Uchida and Kawashima, 2008; Karbach and Kray, 2009; Smith et al., 2009), far transfer of playing videogames to different measures of CC was expected. Transfer from the trained games to unrelated measures of CC was assessed using a cognitive test battery consisting of several tests of updating, shifting, and inhibition.

The improvement of Stop-Signal task and Raven-SPM performance was larger in the videogame group than in the documentary group. Simple effects analyses revealed that performance on neither of these tests improved in the documentary group, whereas the improvement of Stop-Signal task performance in the videogame group was significant and the improvement of Raven-SPM performance was marginally so. Based on these results, it can be concluded that playing cognitive training games online can transfer acquired skills to measures of inhibition and inductive reasoning. The sample under study consisted of relatively high functioning adults. Still, the improvement of Raven IQ entailed an average shift of participants in the videogame group from the 86^th^ to the 90^th^ percentile according to Peck's (1970) norms. The improvement of updating was small too, especially when compared to the extent of age-related decline of working memory (e.g., Baltes and Lindenberger, 1997). At first sight, the improvement of inhibition was substantial, especially in the context of Williams et al.'s (1999) finding that age predicts 5% of SSRT variance across individuals. So, even though inhibition declines with age (Coubard et al., 2011), it is possible to achieve improvements of inhibition on an individual level. This result thus provides evidence supporting the cognitive enrichment hypothesis (Hertzog et al., 2009).

The differential game effect on SSRT qualifies as an example of far transfer (Barnett and Ceci, 2002; Klingberg, 2010), because the videogames were mainly taxing updating, shifting, and inductive reasoning, but not inhibition. Note, however, that inhibition has been argued to form the core of CC. Friedman et al. (2008) performed a behavioral genetics study of CC that included separate measures of updating, switching, and inhibition. Inhibition had a 1.0 loading on the variance in CC, which implies that individual differences in inhibition abilities are closely related to what is common among CC functions.

The current study also, serendipitously, demonstrated a substantial improvement of selective attention in the documentary group, as measured by the UFoV-3, which was absent in the game group. This finding seems at odds with Green and Bavelier's (2003) observations that action videogame players processed more stimulus elements, across a larger visual angle, than non-videogame players. They also observed this difference in a randomized intervention study contrasting action games with Tetris. A tentative solution to this paradox is that a selective attention benefit occurs if an intervention challenges participants to monitor multiple stimuli simultaneously. This was the case in the documentary condition, where participants had to answer quiz questions about details in the documentary, but not in the game condition, as none of the games involved concurrent stimulus presentation. This interpretation is also in line with Green and Bavelier's account of their intervention results.

A critical note concerning the demonstration of transfer to inhibition is in place, however. The differential benefit of the game group for inhibition was partially due to differences between the groups that already existed prior to the intervention (cf. Boot et al., 2011), but that had faded following the intervention. Apparently, the substantial sample size and random assignment of participants had resulted in matched groups in terms of background characteristics, but had not led to sufficient matching of pre-test SSRT. Therefore, there is a risk that the effect of game training was overestimated, so it would be valuable if future studies of game effects could replicate this finding with groups that were matched on pre-test SSRT.

The transfer effects observed in the present study must be interpreted with caution for another reason as well. The current study explored several possible effects of game training. A conservative treatment of the data would therefore require the lowering of alpha to reduce the risk of a Type I error, for example by Bonferroni correction. None of the three interaction effects reported here would survive a Bonferroni correction for testing 10 hypotheses, which would lower the alpha to 0.005, although the simple effect of videogame training on the SSRT would be large enough to survive such an alpha level. The analyses are reported with an uncorrected alpha, however, because the literature on cognitive functions that show transfer of game training among older adults is still rather unexplored. In these circumstances, we find it equally important not to raise the risk of a Type II error.

Statistical shortcomings aside, the present results suggest that Owen et al.'s (2010) and Ackerman et al.'s (2010) negative conclusion regarding transfer of playing online brain training games to CC functions might not necessarily be correct. To a limited extent, the present findings support Basak et al.'s finding that inhibition can be improved by playing videogames and Schmiedek et al.'s (2010) demonstration that inductive reasoning can be improved by practicing basic cognitive tasks. The results from the present study suggest that modest improvements of inductive reasoning can also be achieved by means of playing cognitive training games. A similar partially positive result of games for CC and processing speed was reported by Nouchi et al. (2012).

At the same time, the absence of a benefit of videogame training for two measures of shifting, two measures of working memory span, and two measures of divided attention is reason not to be too optimistic about transfer of game training to higher cognitive functions. This is also the bottom line of the Owen et al. (2010) and Ackerman et al. (2010) studies. There are several points, however, in which the current study was better equipped than previous studies for demonstrating transfer effects of game training.

First, more than half of the participants in Ackerman et al.'s videogame intervention indicated that they did not enjoy playing the videogames. The low compliance to the videogame intervention in Owen et al.'s study also suggests that many participants did not find Owen et al.'s games very engaging either. In the present study, however, most of the participants in the videogame group indicated that they did enjoy playing the videogames. As suggested by Green and Bavelier (2008), motivation is a key condition for transfer to occur. The engaging nature of the videogames used in the present study could thus have facilitated transfer of training.

Second, the composition of the cognitive test battery that is used to assess transfer may confound the results of cognitive training studies. Owen et al. (2010) cognitive test battery, for instance, was restricted to no more than four tests. Owen et al. only obtained measures of updating and inductive reasoning, which may have obscured transfer to other cognitive domains as demonstrated in the present study. Ackerman et al. (2010) included a larger number of tests in their battery of transfer tests, but the test battery mainly comprised measures of perceptual speed and reasoning ability. As a consequence, transfer of training to inhibition, which was found in the present study, could have been overlooked in Ackerman et al.'s (2010) study. Interestingly, several measures of updating and inhibition were included in our test battery, but the positive effect of training on these CC functions could only be detected on one measure of the respective functions. Apparently, the reliability and validity of a cognitive test provides no guarantee for its sensitivity to transfer effects. It can be concluded that the approach of the current study to assess CC functions with an extensive cognitive test battery compensates for the possible insensitivity of some cognitive tests to transfer effects.

Third, the effect of a cognitive training intervention can be underestimated if the control intervention is too effective. The latter might have been the case in Owen et al.'s (2010) study. The control intervention required participants to search for answers to quiz questions on the internet. Participants could have employed and therefore practiced a wide range of strategies for finding answers to the questions. It is impossible to track whether this search caused participants to engage in other cognitively enriching activities. The current study demonstrated that the relatively inactive control condition, consisting of documentary viewing and answering quiz questions already resulted in improved selective attention. It is well possible that Owen et al.'s study presented participants in their control condition with at least the same amount of cognitive challenge.

Finally, not all videogames are created equal (Achtman et al., 2008) and given an individual's stage of cognitive development, one game can be more beneficial for cognitive functions than the other. For example, the cognitive training games used in the present study were very similar to those used in the ACTIVE study (Ball et al., 2002). Preliminary evidence for far transfer of cognitive training games was found in the present study but only near transfer was found in the ACTIVE study. The different extent of transfer in the ACTIVE study may be explained by the additional focus on learning to use specific strategies to perform the training tasks. Relying on a set of fixed strategies to cope with demands of a task at hand could have reduced the degree to which participants needed to exert CC during training. Thus, even though videogames of a similar genre were investigated in the present study and the ACTIVE study, a small difference between the intervention programs may be responsible for the different patterns of transfer that were observed.

In conclusion, the present study lends modest support to the notion that playing cognitive training games improves untrained CC functions in older adults. Since CC functions facilitate adaptive behavior in various contexts, improved CC can be expected to help older adults to overcome cognitive challenges in their daily routines. Videogames provide an entertaining and thus motivating tool for improving CC functions and they have other practical advantages as well. Videogames do not require physical well-being and mobility of the participant as much as physical exercise interventions, although these seem to be more effective in buffering decline of CC (cf. Colcombe and Kramer, 2003). Additionally, videogames are not expensive to administer as compared to interventions supervised by a therapist. Videogames come in forms far more complex than cognitive tests usually studied by cognitive psychologists. The present study suggests that the videogames should not be dismissed as a cognitive training tool, but that we are just beginning to understand how playing videogames influences cognitive functions.

Even within the homogeneous sample of older adults that participated in the present study, some participants benefited more from playing the videogames than others. A variety of factors may be responsible for individual differences in sensitivity to cognitive training. For instance, recent findings from our lab indicate that inter-individual genetic variability modulates transfer of training to untrained tasks (Colzato et al., 2011). Therefore, caution concerning the interpolation of aggregate data to individuals is advised, and individual differences in cognitive training outcomes are an important topic to be addressed in future studies.

The artwork of the games we presented here was not nearly as advanced and capturing as commercial off-the-shelf games, and that applies to most studies of game training. Conversely, commercial enhancement games are only seldom designed on the basis of cognitive insights, nor tested for their effectiveness. Given that the creative industry and academic research are only just starting to inspire each other's work, these first modest demonstrations of cognitive enhancement by games may only be scratching the surface of its full potential.

### Conflict of interest statement

The authors declare that the research was conducted in the absence of any commercial or financial relationships that could be construed as a potential conflict of interest.
